# Catalytic CVD Synthesis of Carbon Nanotubes: Towards High Yield and Low Temperature Growth

**DOI:** 10.3390/ma3114871

**Published:** 2010-11-01

**Authors:** Arnaud Magrez, Jin Won Seo, Rita Smajda, Marijana Mionić, László Forró

**Affiliations:** 1 Laboratory of Complex Mater Physics, Ecole Polytechnique Fédérale de Lausanne, 1015 Lausanne, Switzerland; E-Mails: rita.smajda@epfl.ch (R.S.); marijana.mionic@epfl.ch (M.M.); laszlo.forro@epfl.ch (L.F.); 2 Center for Research on Electronically Advanced Materials, Ecole Polytechnique Fédérale de Lausanne, 1015 Lausanne, Switzerland; 3 Department Metallurgy and Materials Engineering, Katholieke Universiteit Leuven, 3001 Heverlee, Belgium; E-Mail: maria.seo@mtm.kuleuven.be

**Keywords:** carbon nanotubes, catalytic chemical vapor deposition, catalyst, catalyst support

## Abstract

The catalytic chemical vapor deposition (CCVD) is currently the most flexible and economically attractive method for the growth of carbon nanotubes. Although its principle is simple, the precisely controlled growth of carbon nanotubes remains very complex because many different parameters influence the growth process. In this article, we review our recent results obtained on the synthesis of carbon nanotubes via CCVD. We discuss the role of the catalyst and the catalyst support. Our recent results obtained from the water assisted growth and the equimolar C_2_H_2_-CO_2_ reaction are also discussed. Both procedures lead to significantly enhanced carbon nanotube growth. In particular, the latter allows growing carbon nanotubes on diverse substrate materials at low temperatures.

## 1. Introduction

The catalytic chemical vapor deposition (CCVD) is currently the most viable process for the synthesis of carbon nanotubes. Many research groups have successfully attempted to accurately control the physical form of the carbon nanotubes produced [1,2,3,4,5,6, and references therein]. In particular, the influence of numerous growth parameters on the resulting nanotubes characteristics, such as diameter, length, number of graphene layers, defect density *etc.*, has been studied [7,8]. At present, several basic aspects of the growth mechanisms have been established [9,10,11,12,13,14, and references therein]: the catalytic decomposition of the carbon precursor molecules on the surface of the supported metal catalyst is followed by diffusion of the carbon atoms produced either on the surface or in the metal particles. The growth temperature, as well as the particle size, determines the limit of carbon solubility in the metal particle. Super-saturation of the metal results in solid carbon precipitation and the subsequent formation of the nanotubes structure. Two different growth mechanisms can occur depending on the catalyst-support interaction. The tip-growth, where the catalyst is lifted off the support while carbon nanotube grows, takes place when the metal-support interaction is weak. In contrast, the root growth process occurs when the metal-support contact is preserved and the catalyst particles remain on the support during the carbon nanotube growth.

Of specific interest has been the carbon nanotubes production efficiency at lower temperatures in order to enable direct integration of carbon nanotubes into devices [15,16,17,18]. In particular, catalyst modification by pre-growth chemical activation [17,18,19] and/or prevention of catalyst poisoning, for instance by introduction of an etching agent which prevents the encapsulation by amorphous carbon precipitation, have been studied [20,21]. Recent outstanding results have demonstrated that the presence of a small and controlled amount of oxygen containing species, in addition to the carbon source, dramatically improves the yield of the reaction [20,21]. The effect observed is not dependent on the carbon source or on the processing method. It is assumed that the presence of H_2_O and CO_2_ during the growth etches amorphous carbon effectively away and prevents catalyst particle encapsulation by amorphous carbon [22]. Very recently, it was discovered that other additives (e.g., a sulfur-containing compound, thiophene) improved the density of carbon nanotubes indicating that the nucleation and growth of carbon nanotubes can be influenced by novel chemical reactions [23].

The growth of carbon nanotubes still remains complex. In this paper, we review our studies on the synthesis of carbon nanotubes via the CCVD process. We first examine the influence of the catalyst, as well as the influence of the support material. Subsequently, the effect of adding water or CO_2_ to the process gas mixture is discussed with respect to the carbon nanotube growth efficiency. Our results demonstrate the “special” case of CaCO_3_ which releases CO_2_ by its thermal decomposition and actively influences the growth of carbon nanotubes. The interaction between C_2_H_2_ and CO_2_ significantly promotes the carbon nanotube growth and even allows the growth of carbon nanotubes in thermodynamically and/or kinetically unfavorable conditions. We also produced carbon nanotubes using the water-assisted process and compared the bending modulus of the carbon nanotubes obtained from the water-assisted growth and of those produced from the equimolar C_2_H_2_-CO_2_ reaction. The latter provides the possibility to significantly decrease the growth temperature, as well as to obtain carbon nanotubes growth on various substrate materials.

## 2. Results and Discussion

Most of the growth studies we have performed so far used catalyst particles supported by powder substrates. Due to the higher surface area, powder support leads to higher catalyst efficiency and higher carbon nanotubes yield. Therefore, we rely on the results obtained from powdered support materials in order to discuss the yield and growth efficiency. However, the results are also valid for the carbon nanotubes growth on flat substrates. As we recently demonstrated [24], vertically aligned growth of carbon nanotubes can be obtained on Si with a buffer layer, as well as powder supports pressed to a pellet. As our results demonstrate, the choice of the catalyst and that of the support strongly influence the carbon nanotubes growth. Nevertheless, involving additional species, such as water and CO_2_, into the reaction gas can significantly enhance the catalytic activity.

### 2.1. Catalyst

Generally, nano-sized transition metal particles, e.g., nickel, iron, cobalt, molybdenum and copper, have been successfully used in CCVD either in oxide or metallic forms or as mixtures [7,8]. A very large amount of papers report on the growth of carbon nanotubes, using different compositions, size, preparation methods and crystallographic orientation. A general overview of the different catalysts can be found in the review papers [7,8,9]. The most important property of the metal particles with regard to carbon nanotube formation is their ability to catalytically decompose gaseous carbon-containing molecules. The solubility of carbon in catalyst particles is size-dependent. As Moisala *et al.* [7] highlighted in their review, the solubility of carbon in iron and nickel significantly increases for metal particles with a diameter of less than approximately 10 nm. Seidel *et al.* investigated the difference of the carbon nanotubes growth behaviors for Fe, Co, and Ni catalysts [16]. They observed that the order of the lowest growth temperatures agrees with the order of the bulk melting points of the three transition metals (Ni, 1,450 °C; Co, 1,490 °C; Fe, 1,540 °C). For binary compounds, e.g., cobalt–molybdenum [25], iron–molybdenum [26] and iron–cobalt [27,28], the yield of carbon nanotubes has been observed to increase significantly. However, the precise catalyst composition has a great influence on the final product. We have investigated Fe_1-x_Co_x_ and Fe_1-x_Ni_x_ alloys for the growth of carbon nanotubes [28,29].

#### 2.1.1. Catalyst composition

**Figure 1:**
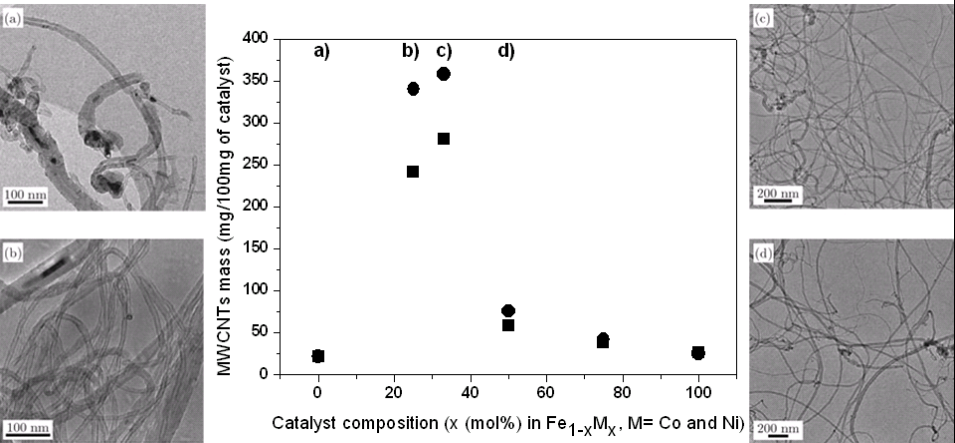
Effective mass of the obtained multi-walled carbon nanotubes after purification as a function of the catalyst composition Fe_1-x_M_x_ with M = Co (■) and Ni (●). The TEM images show representative carbon nanotubes obtained for (**a**) x = 0 mol%, (**b**) x = 25 mol%, (**c**) x = 33 mol% and (**d**) x = 50 mol% Fe_1-x_Co_x_ catalyst.

Fe1–xCox alloys are among the most efficient catalysts for the synthesis of multi-walled carbon nanotubes [27,28]. The resulting yield, as well as the nanotubes characteristics, strongly depend on the parameter x. The highest yield, the narrowest diameter distribution, and the least defect density in the nanotubes graphitic structure, are obtained at x = 33 mol% [28]. As highlighted in Figure 1, the catalyst efficiency increases almost 100 times by changing x from 0 to 25 mol% for growth temperature at 700 °C. For x = 33 mol%, the increase is even slightly higher but exceeding 33 mol% results in a sudden drop to a catalyst efficiency comparable to that of the monometallic Fe catalyst (x = mol 0%). Increasing x even more does not change the yield significantly and keeps the efficiency similarly low. Thus, the metallic alloy Fe_2_Co is the most active catalyst in the Fe1–xCox compound with a catalytic activity for carbon nanotubes growth more than 100 times higher than that of monometallic catalysts Fe and Co [28].

The catalyst efficiency also determines the carbon nanotube diameter, as well as the amount of catalyst particles present in the product. As can be seen in the transmission electron microcopy (TEM) images in Figure 1, carbon nanotubes reveal high diameter distribution with diameters ranging from 10 nm to 50 nm when the composition strongly deviates from x = 33 mol%. Moreover, many catalyst particles can be found as embedded particles. In contrast, for x = 33 mol% carbon nanotubes with diameter of 15 ± 2 nm are observed without any presence of enclosed catalyst particles. In order to determine the precise structure and the composition of the catalyst, x-ray powder diffraction (XRPD) was performed on supported catalysts under various conditions: After annealing at the carbon nanotubes growth temperature under (i) pure N_2_ and (ii) N_2_ mixed with acetylene (same conditions as for the carbon nanotubes growth) [28]. According to these measurements, a single-phase catalyst forms at x = 33 mol% after 10 min of annealing under N_2_. This is identified as the spinel phase Fe_2_CoO_4_. A deviation from this optimum composition leads to the formation of Fe_2_Co_3_ and CoO phases for x < 33 mol% and x > 33 mol%, respectively. Additional annealing under N_2_ mixed with C_2_H_2_ reduces the oxide. Pure Fe_2_Co is obtained for x = 33 mol% whereas Fe_3_C and Co are formed for pure Fe and Co catalysts, respectively. In an intermediate composition, two phases are found, namely Fe_2_Co and Fe_3_C or Co depending on the Fe- or Co-rich composition. Their fraction is determined by the precise composition. The TEM micrographs shown in Figure 1 clearly demonstrate that any deviation from the x = 33 mol% composition results in the presence of the secondary phase Fe_3_C or Co, which leads to high density of enclosed particles in carbon nanotubes and significantly reduces the resulting carbon nanotubes yield.

Similarly, we have studied the Fe_1-x_Ni_x_ alloys family. Again a spinel phase is formed for x = 33 mol%, namely Fe_2_NiO_4_. The corresponding Fe_2_Ni catalyst is more active than pure Ni and pure Fe. The mass of carbon nanotubes produced over Fe_2_Ni is more than 10 times higher than over pure Fe or Ni catalyst [29]. As can be seen in Figure 1, the resulting carbon nanotube mass obtained from 100 mg catalyst is even higher for Fe_1-x_Ni_x_ at 25 and 33%. Our preliminary results indicate that the multi-walled carbon nanotubes obtained from Fe_2_Ni are more defective than those obtained from Fe_2_Co indicating that the growth condition still needs to be optimized. Nevertheless, the recent result reported by Chiang and Sankaran [31] indicate that the chirality distribution of as-grown carbon nanotubes can be altered by varying the composition of Fe_1-x_Ni_x_ nanocatalysts. By precisely tuning the nanocatalyst composition at constant size they observed a link between the composition-dependent crystal structure of the nanocatalysts and the resulting nanotube chirality.

#### 2.1.2. Catalyst drying process

The drying process of the catalyst prior to the carbon nanotubes production is an important step. When catalysts are dried by heating, large agglomerates of catalysts are formed which cannot be broken into small particles by additional mechanical grinding. Consequently, when the catalyst is introduced into the reactor, only the outer surface of the large agglomerates is exposed to the reaction gas. Therefore, the catalyst efficiency is strongly limited. In addition, the multi-walled carbon nanotubes produced are of very poor quality with a broad diameter distribution. The catalyst efficiency can be quantified as the mass of purified multi-walled carbon nanotubes produced, divided by the mass of supported catalyst introduced. Typically, catalysts dried by heating lead to a catalyst efficiency of around 1.2. To be precise, 120 mg of multi-walled carbon nanotubes are produced from 100 mg of catalyst supported by CaCO3. Thus, during the synthesis, 18% of acetylene is converted into multi-walled carbon nanotubes.

As we recently reported, freeze drying of the catalyst is a favorable alternative approach which avoids agglomeration of the supported catalyst particles during the sublimation of water [30]. To be precise, the prepared catalyst suspension is frozen by dropping into liquid nitrogen. Once collected, it is subsequently placed in a freeze drying chamber, where sublimation occurs by raising the temperature while keeping the vapor pressure below 5 mbar.

**Figure 2:**
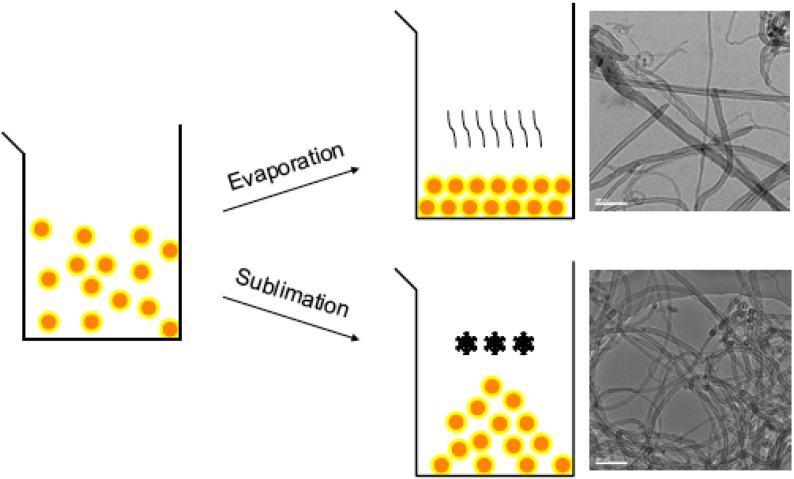
Schematic drawing illustrating the freeze drying, compared to the hot drying, process. Agglomeration of the supported catalyst particles is avoided when freeze dried. TEM micrographs show representative carbon nanotubes produced from these two drying processes (scale bar is 100 nm).

Using the freeze drying method, it was possible to increase the acetylene conversion for carbon nanotubes growth to 85% (using Fe_2_Co catalysts loaded on CaCO_3_ particles as support). This corresponds to a catalyst efficiency of 5.6, which means 560 mg of multi-walled carbon nanotubes are produced from 100 mg of supported catalyst. Thus, freeze drying is more than four times more efficient than heating. The produced multi-walled carbon nanotubes exhibit a mean diameter of about 11 nm with a diameter distribution of about ±6 nm. Consequently, the catalyst efficiency dramatically increases and high quality multi-walled carbon nanotubes are produced with a narrow diameter distribution (see the TEM image in Figure 2).

### 2.2. Support

Various support materials have been tested for the CCVD synthesis of carbon nanotubes. Most commonly, Al_2_O_3_ and SiO_2_ have been used [7,8]. MgO has been reported to be appropriate for the growth of single-walled carbon nanotubes. A general prerequisite for a support material is thermal and chemical stability under the synthesis conditions. Consequently, direct growth on Si [32] or a metal substrate [33,34] has been difficult because of the diffusion of the catalyst material into the support and formation of silicides or alloys upon heating to the carbon nanotube growth temperature. Additional properties of importance for efficient carbon nanotube growth have been the surface area and porosity. Chemical and/or physical interaction may take place between the support and the catalyst material. Physical interactions (e.g., van der Waals and electrostatic forces, together with obstruction of catalyst particle movement on the support surface due to surface roughness) can reduce the thermally driven diffusion and sintering of metal particles on the support surface. This leads to stabilization of the catalyst particle size distribution. The chemical interactions between the catalyst particles and the support surface can lead to limited particle growth due to decreased particle mobility. By *in situ* photoelectron spectroscopy it has been demonstrated that the catalyst–substrate interaction during the CCVD process determines the chemical oxidation state of the catalyst, which affects the carbon nanotube characteristics [35,36]. Al_2_O_3_ has been generally observed to be a superior support compared to SiO_2_, TiN or TiO_2_ [36,37]. The stronger chemical interaction between Al_2_O_3_ and the metal catalyst has been appointed to the oxidation process occurring at the catalyst–Al_2_O_3_ interface.

Support materials such as Al(OH)_3_ and CaCO_3_ also have shown to positively influence the carbon nanotube growth process by their decomposition [28,38,39]. We have systematically studied the carbon nanotube synthesis using CaCO_3_ and other alkaline earth carbonates.

#### 2.2.1. Alkaline earth carbonates

CaCO_3_ is one of the most efficient supports used for the synthesis of multi-walled carbon nanotubes [38,40,41]. In our previous paper, we demonstrated that Fe_2_Co supported by CaCO_3_ results in an efficient selective formation of carbon nanotubes [28,42]. The carbonate is also favorable because of its simple removal allowing a simple one-step purification of carbon nanotubes without any perceivable damage of the carbon nanotube structure. Both metallic particles and catalyst support can be dissolved in diluted mineral acids (e.g., HNO_3_, HCl) [40]. Nevertheless, one of the most exceptional characteristics of CaCO_3_ is its decomposition. The CaCO_3_ stability is ruled by a dynamic equilibrium of the decomposition reaction CaCO_3_ ↔ CaO + CO_2_ in the temperature range of 600 to 800 °C. Consequently, CO_2_ is available as free gas or bound to the support as CaCO_3_ in a ratio that depends on the temperature. In a first approximation, the partial pressure of CO_2_ and the average decomposition rate of the carbonate can be deduced from thermogravimetric analysis (TGA) of CaCO_3_ [28,43].

**Figure 3:**
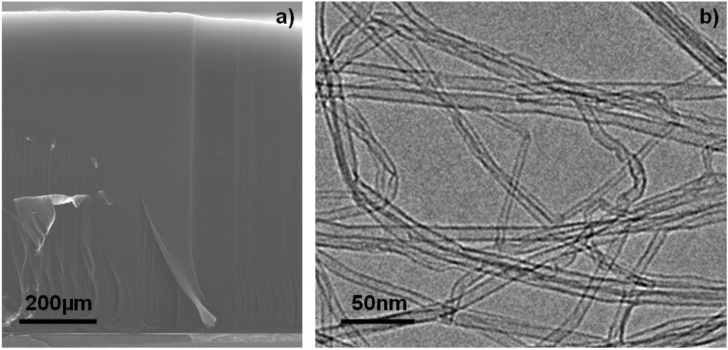
(**a**) Cross-sectional SEM image of a carbon nanotubes forest with a height of about 1mm grown by the water assisted CCVD growth process. (**b**) Representative TEM image of the carbon nanotubes produced at 750 °C. Carbon nanotubes are clean, free of amorphous carbon and catalytic particles.

In Figure 3, the maximum carbon nanotubes yield obtained from Fe_2_Co supported by CaCO_3_ is shown as a function of the decomposition rate of CaCO_3_. In the temperature range between 640 and 680 °C about 5% of CaCO_3_ decomposes. The highest yield was obtained in this range: About 350 mg of multi-walled carbon nanotubes are produced from 100 mg of Fe_2_Co/CaCO_3_ catalyst in about 30 minutes. This corresponds to a conversion of about 54% of acetylene. By means of a quadrupolar mass spectrometer (QMS), the precise CO_2_(g)/C_2_H_2_ ratio at 660 °C was measured to 1:100 and was found to be stable over the entire period of the carbon nanotubes growth. A small variation in the decomposition rate of CaCO_3_, which is controlled by the variation of the growth temperature, can decrease the yield dramatically. For instance, an increase of 40 °C can lead to a 40% reduction of the maximum yield indicating how strongly the CO_2_ content influences the reaction.

We also examined other alkaline earth carbonates as supports [28]. Carbon nanotubes are obtained in all samples but the yield strongly varies. The highest yield is obtained with CaCO_3_, which has the decomposition temperature closest to the carbon nanotube growth temperature. For MgCO_3_, the decomposition temperature (300 °C) is significantly lower than the carbon nanotube growth temperature. Therefore, the support fully transforms into MgO, already before the carbon nanotubes growth, and CO_2_ cannot contribute to the growth process. For SrCO_3_ and BaCO_3_, the decomposition temperature is substantially higher, 970 °C and 1,320 °C, respectively. Hence, CO_2_ groups maintain in the carbonate and do not react with acetylene.

CaCO_3_ is an excellent support, not only for the production of high-yield multi-walled carbon nanotubes, but also for that of single-walled CNTs [44]. We applied a combustion method in order to produce well dispersed catalysts, with reduced size and with a control of the diameter. The combustion technique is typically used for the production of highly dispersed powder-like materials and exploits an exothermic, very rapid redox-type chemical reaction [45,46]. However, acetylene was found to be a too rich carbon source and extremely difficult to control. By reducing the acetylene flow, only double-walled carbon nanotubes could be obtained. By exposure to ethylene and methane, high density of single-walled carbon nanotubes with a diameter distribution of 1.1 ± 0.3 nm was produced [44].

#### 2.2.2. Size of support particles

For an efficient carbon nanotubes synthesis, also the particle size of the support plays a role. As demonstrated in our previous work [30], calcite powders with very low specific surface area (S_BET_ < 1 m^2^/g) can mechanically be grinded in order to reduce the particle size and to increase the specific surface area. The resulting average particle size was 50 nm with a diameter distribution of about ±25 nm. The specific surface area was about 5.6 m^2^/g as derived from granulometry powder characterization with Malvern Mastersizer S spectrometer. We compared this powder with a second one (from Calofort U), which had a specific surface area of 4.6 m^2^/g and particle size of about 100 nm with a broad diameter distribution of ±60 nm.

At first, catalyst particles are produced from Fe_2_Co nanoparticles using the second type of CaCO_3_ particles with a larger particle size and slightly lower surface area, applying the free drying mentioned in Section 2.1.2. About 44% of acetylene was converted into the formation of multi-walled carbon nanotubes. This corresponds to a catalyst efficiency of 2.9. When fine ground CaCO_3_ particles from the first type are employed, the conversion increases to about 85%, which corresponds to a catalyst efficiency of 5.6 (560 mg of multi-walled carbon nanotubes are produced from 100 mg of supported catalyst). This result clearly illustrates that the acetylene conversion into carbon nanotubes formation can be controlled by the support particle size.

The support particle size does not only impact the carbon nanotubes production yield but also the diameter distribution of the resulting carbon nanotubes. The mean diameter of the multi-walled carbon nanotubes obtained from the second type of CaCO_3_ particles is about 14 ± 4 nm, whereas the multi-walled carbon nanotubes synthesized from the fine ground CaCO_3_ exhibit a smaller average diameter of 11 ± 6 nm. The effect of the support particle size can be explained by the triple point junction area, where nanotubes growth stems. It corresponds to the location where the reaction between CO_2_ (originating from the CaCO_3_ support) and C_2_H_2_ is catalyzed by Fe_2_Co particles [43]. When catalyst is deposited on materials with a large surface area, Fe_2_CO is distributed over a large surface and the co-precipitation process yields small particles. Small particles have larger relative interface to the support with respect to their volume. Hence, the relative interface area (being the active area for the chemical reaction) is larger for small particles and consequently the catalyst efficiency is enhanced, leading to larger quantity of carbon nanotubes being produced.

### 2.3. CCVD Process

CCVD is a simple and economic technique for synthesizing carbon nanotubes at low temperature (300–1,200 °C) and ambient pressure. It is versatile and can utilize a variety of carbon sources in any state (solid, liquid, or gas); enables the use of various substrates; and allows carbon nanotube growth in a variety of forms, such as powder and films. Therefore this technique is ideally suited for producing arrays of individual, or a mat of aligned carbon nanotubes, as well as for a desired architecture of a nanotube device. Commonly used gaseous carbon sources have been methane, acetylene and carbon monoxide. In the case of liquid carbon sources, an alcohol, such as methanol and ethanol, is heated in a flask and purged with an inert gas in order to carry the vapor into the reaction furnace. Alcohol-assisted growth has yielded single-walled carbon nanotubes at a relatively low minimum temperature of about 550 °C [15]. The most recent breakthroughs in the CCVD synthesis of carbon nanotubes represent the water-assisted growth [20] and the equimolar CO_2_-C_2_H_2_ reaction [43].

#### 2.3.1. Water assisted growth

Pioneered by Hata *et al.* [20], the so-called “super growth CVD process” based on the introduction of traces of water has been considered as one of the most efficient growths resulting in dramatically enhanced carbon nanotubes growth. Water is believed to extend the catalyst lifetime by etching the amorphous carbon deposit on the surface of the catalyst. In the optimum growth conditions, more than 85% of the catalyst nanoparticles are active [47]. The growth kinetics is also strongly extended so that millimeter high carpets of vertically aligned carbon nanotubes can be grown in a few minutes [22]. By optimizing the growth conditions, 1 cm high dense mat of carbon nanotubes forest was demonstrated very recently [48].

**Figure 4:**
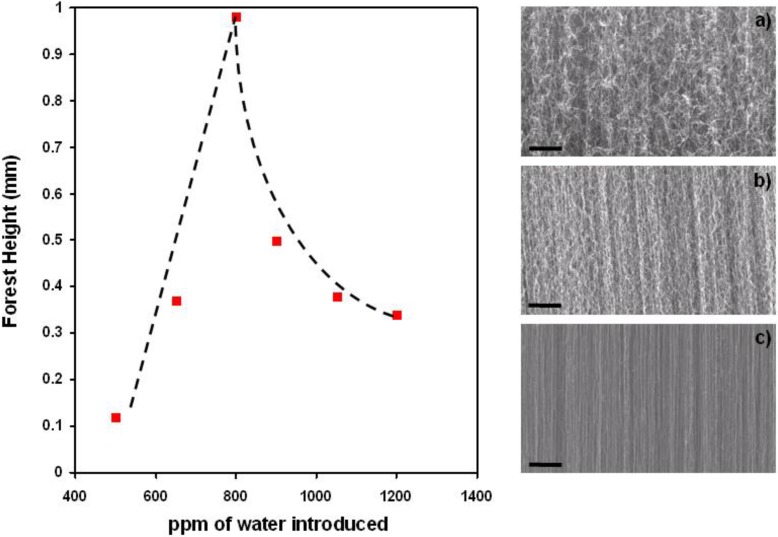
The height of the resulting carbon nanotubes forests strongly varies with the ppm of water introduced into the reactor. The SEM micrographs obtained at (**a**) 500 ppm, (**b**) 800 ppm and (**c**) 1,200 ppm, illustrate that the carbon nanotubes alignment, as well as their density, significantly changes with the amount of added water. Whereas the thickest carpet is produced at 800 ppm, the best alignment is obtained at 1,200 ppm. Dashed lines guide the eyes. Scale bars are 2 µm.

We have produced carbon nanotube forests in growth conditions [49] slightly different to those applied in [20,22,47,50]. In particular, gas phase composition in the reactor is enriched with ethylene and water while keeping other growth parameters similar to the ones reported in [20,22,47,50]. The obtained forest height is about 950 µm produced in 30 min, comparable to the previous reported results. TEM studies of the as-grown carbon nanotubes reveal that the grown carbon nanotubes have a narrow diameter distribution. Single-walled, as well as multi-walled, carbon nanotubes with small number of walls (2–5 walls) are found. The average outer diameter is about 7–8 nm. Carbon nanotubes are clean and free of amorphous carbon as well as catalytic particles indicating that high purity carbon nanotubes are produced by the water assisted CCVD process (Figure 3). In particular, the absence of amorphous carbonaceous materials suggests the cleansing role of water during the carbon nanotubes growth. By precisely varying the gas phase composition during the CCVD process, we obtain carbon nanotube forests with different heights. As illustrated in Figure 4, the maximum height is obtained by introducing 800 ppm of water. This corresponds to a H_2_O/C_2_H_4_ ratio of about 1/1000, which is in agreement with the results obtained by the group of Hata [22]. A slight deviation from this value dramatically decreases the forest height indicating that the ethylene/water ratio is one of the most critical parameters to control in order to exploit the advantages of the water assisted CCVD process. Moreover, scanning electron microscopy (SEM) micrographs of forests produced with different water content (see Figure 4) demonstrate that also the carbon nanotubes alignment strongly depends on the H_2_O/C_2_H_4_ ratio.

The best alignment is not found in the forest with the highest height, but in that produced with the highest water content of 1,200 ppm. The alignment in carbon nanotube forest is caused by the strong van der Waals interaction between individual tubes. When carbon nanotubes density is low, van der Waals interaction is low and carbon nanotubes grow randomly and curved so that the carpet height does not reflect the real carbon nanotubes length (Figure 4a). When the water content is raised, alignment improves, because raising the H_2_O/C_2_H_4_ ratio increases the number of active catalytic particles and consequently the density of the forest. However, diffusion of feedstock gas to catalyst particles becomes more and more difficult with the increasing density, as well as with the dense forest thickness. The growth finally stops because of the obstruction of the gas flow. As can be seen in Figure 4b, for the water content of 800 ppm, the highest forest thickness is obtained although the alignment is rather poor. This means that the poor alignment provides space for ethylene to continuously penetrate down to the catalytic particles. When water content is raised more, all catalytic particles are activated, and a very dense mat of almost perfectly aligned carbon nanotubes is produced. However, ethylene penetration through the carpet is reduced and therefore carpet height is significantly reduced (Figure 4c). Yasuda *et al.* [48] demonstrated very recently that this limitation could be circumvented by controlling the gas flow direction. Using a gas shower system, providing direct delivery of gases from the top of the forest, they achieved higher carbon nanotubes forests growth stability, uniformity, reproducibility, carbon efficiency (32%), and catalyst lifetime. With this improved growth, they could synthesize a 1 cm tall forest with 1cm x 1cm size and demonstrated the scalability of water-assisted CCVD to A4-size.

Mechanical bending modulus measurements of individual carbon nanotubes indicate that very high quality carbon nanotubes are produced by the water assisted CCVD process. The obtained bending modulus is significantly higher compared to the values measured from the carbon nanotubes produced by the conventional CCVD process [51]. The values (390–631 GPa) are close to the ones obtained from the best CCVD grown carbon nanotubes [52]. Hence, the advantage of the presence of water is twofold: (i) its weak oxidizing effect prevents amorphous carbon deposition and (ii) it does not produce extended damages in the graphitic structure of the carbon nanotubes, even when water concentration as high as 1,200 ppm is used.

**Figure 5:**
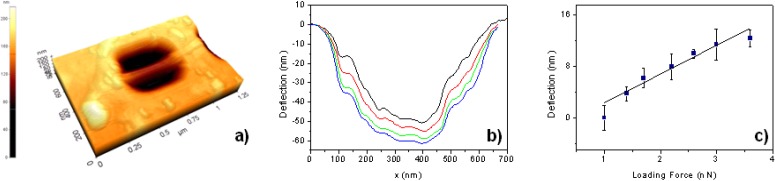
(**a**) Representative atomic force microscopy (AFM) image of a nanotube suspended over the hole of an alumina membrane for the individual mechanical bending modulus measurements. (**b**) Representative deformation profile of a suspended carbon nanotube and (**c**) deflection *vs.* loading force derived from the deformation profile. The slope is proportional to the bending modulus of the carbon nanotubes.

**Table 1:** Mean, maximum and minimum, bending modulus (mean, max. and min. E_b_) of carbon nanotubes produced by the water assisted growth and the equimolar reaction.

	Mean E_b_ (GPa)	Max. E_b_ (GPa)	Min. E_b_ (GPa)
Water assisted grown carbon nanotubes	570	1,200	240
Equimolar grown carbon nanotubes	390	1,040	56

In Table 1 the mean, the maximum, and the minimum, bending modulus are summarized for carbon nanotubes grown via the water assisted synthesis and the equimolar C_2_H_2_-CO_2_ reaction. The latter CCVD route will be described in detail in the following Section 2.3.2. The main difference observed in the mechanical properties of the carbon nanotubes produced by the equimolar reaction process and by the water assisted method is the smaller range of bending modulus variation for carbon nanotubes produced by the water assisted growth process. This phenomenon is based on the fact that carbon nanotubes, produced by the water assisted growth process, exhibit a smaller average diameter with a narrower diameter distribution. In the case of the equimolar process, carbon nanotubes were produced using CaCO_3_ nanoparticle support, generally leading to a larger carbon nanotube diameter and a broader diameter distribution compared to carbon nanotubes from the water assisted growth.

#### 2.3.2. Equimolar C_2_H_2_-CO_2_ reaction

Inspired by the carbon nanotubes growth using CaCO_3_ support that produces CO_2_ by thermal decomposition, we studied the precise effect of CO_2_ on the carbon nanotubes growth by adding CO_2_ gas into the reactor. For these studies, MgO was selected as catalyst support because MgO does not form a carbonate at temperatures higher than 300 °C (see Section 2.2.1). Consequently, carbon nanotubes synthesis at temperatures higher than 300 °C excludes the CO_2_ contribution from the support and enables monitoring of the carbon nanotubes growth with the precise control of the added amount of CO_2_ gas. At 680 °C, the carbon nanotubes yield obtained as a function of the CO_2_/C_2_H_2_ ratio reveals a maximum at CO_2_/C_2_H_2_ = 1 [43,44]. Any deviation from this optimum gas-phase composition does not only decrease the carbon nanotubes yield but also leads to the production of amorphous carbon. In the absence of CO_2_ no carbon nanotubes are produced. It has to be mentioned that carbon nanotubes growth using MgO typically requires high temperature (>750 °C). Thus, C_2_H_2_-CO_2_ reaction enables synthesis of carbon nanotubes in conventionally unfavorable conditions.

We have very recently discovered that the equimolar C_2_H_2_-CO_2_ reaction can be applied to many different support systems resulting in a flexible, highly reproducible and high yield synthesis of carbon nanotubes [24]. The C_2_H_2_-CO_2_ reaction leads to oxidative dehydrogenation reaction of acetylene, which has been widely used in olefin industry for the production of unsaturated hydrocarbons [54]. However, to our knowledge this process has never been considered for the synthesis of carbon nanotubes.

**Figure 6:**
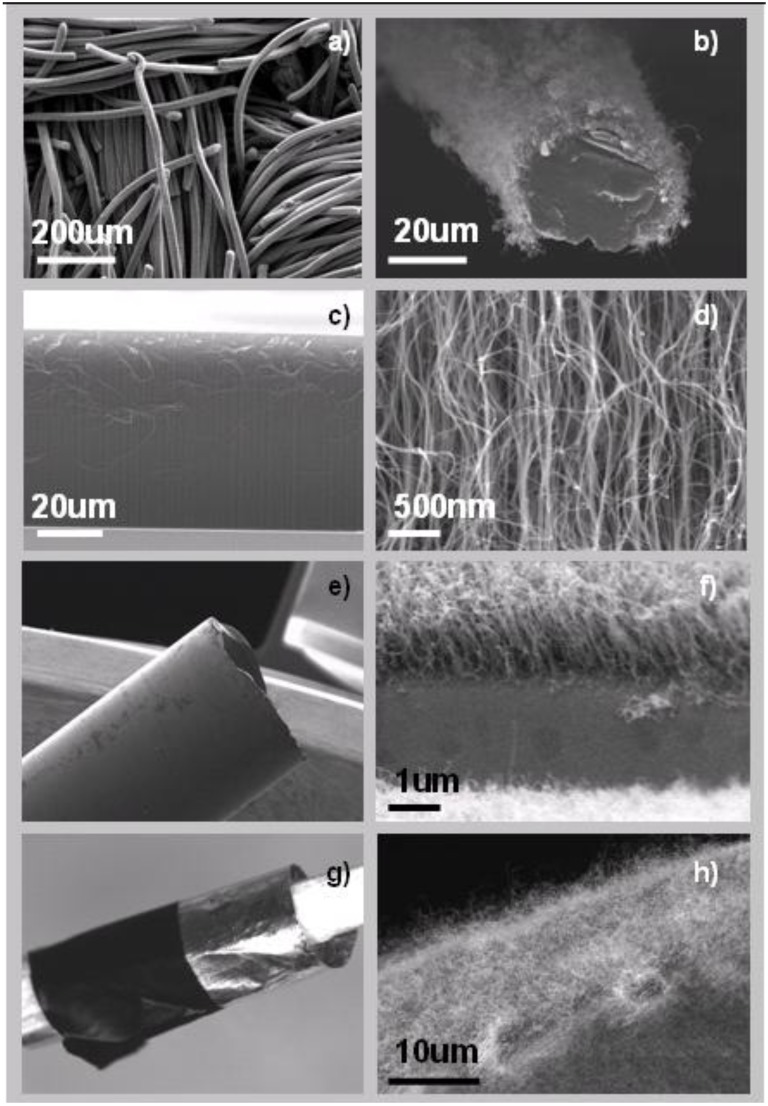
Carbon nanotubes grown by the equimolar reaction over (**a,b**) carbon fibers, (**c,d**) carbon nanotubes forest over Fe_2_Co particles supported by an alumina thin film, (**e**) and (**f**) carbon nanotubes on glass, (**g**) and (**h**) carbon nanotubes on aluminum foil.

Figure 6 demonstrates representative growth of carbon nanotubes produced by the oxidative dehydrogenation reaction of C_2_H_2_ with CO_2_ on a broad range of support materials including carbon fibers, alumina, glass and aluminum foil. These materials are decorated prior to the growth with Fe based-metallic nanoparticles (Fe_2_Co or Fe) by a simple co-precipitation process (see Section 3 for details). For all support materials, the produced carbon phase entirely consists of carbon nanotubes, if the precise equimolar stoichiometry between C_2_H_2_ and CO_2_ was applied. As observed by TEM, carbon nanotubes are multi-walled and of high crystalinity without any indication of amorphous carbon. Similar structural characteristics are observed for all samples independent of the support material applied [24, see also supporting information].

**Figure 7:**
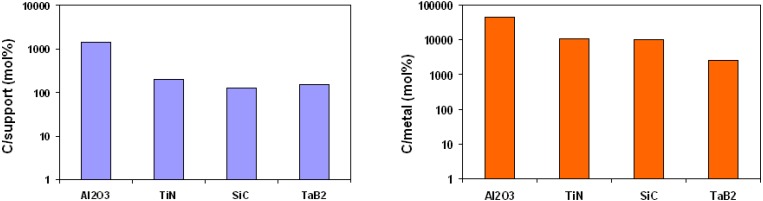
Efficiency of the carbon nanotubes produced by the equimolar reaction presented as (a) the ratio between the mass of carbon nanotubes obtained and the amount of support and (b) the ratio between the mass of carbon nanotubes obtained and Fe_2_Co catalyst introduced into the reaction chamber.

In Figure 7, the resulting carbon nanotubes yield is presented as the ratios C/support and C/metal obtained for various support materials including oxides, borides, nitrides and carbides. The numbers vary with the different support material. However, it has to be stated that on all these supports, the obtained carbon nanotubes yield is at least 10 times higher than without the oxidative dehydrogenation reaction.

The oxidative dehydrogenation reaction can proceed along two overall chemical mechanisms as we proposed previously [24,42]:

C_2_H_2_ + CO_2_ → 2C + H_2_O + CO (1)

C_2_H_2_ + CO_2_ → C + H_2_ + 2CO (2)

Besides these two mechanisms, the thermal decomposition of acetylene can occur. However this process is kinetically limited at low temperatures, whereas the reactions (1) and (2) can spontaneously take place. In order to identify the precise chemical reaction involved, residual gas composition was analyzed in the exhaust during the synthesis. In particular, the partial pressures of the main reaction products (H_2_O and CO) were monitored by means of quadrupolar mass spectroscopy (QMS).

**Figure 8:**
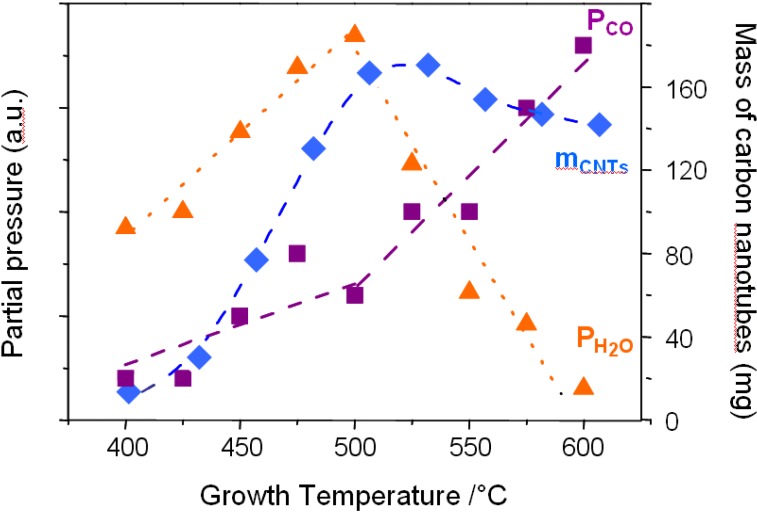
Evolution of the partial pressure of CO and H_2_O, as well as the mass of carbon nanotubes produced as a function of growth temperature. Catalyst applied is Fe_2_Co supported by Nb_2_O_5_. At 500 °C, a transition occurs for both parameters, as well as for the mass of the carbon nanotubes produced.

Figure 8 shows the partial pressures measured during the synthesis of carbon nanotubes using Fe_2_Co supported by Nb_2_O_5_. The amount of water and CO produced clearly undergo temperature dependence. At 500 °C, a sudden change occurs where the water partial pressure, which was constantly increasing, abruptly decreases. Simultaneously the CO content increases. This significant change in the reaction gas composition at 500 °C can be explained by the change of reaction. Below 500 °C, a high amount of H_2_O is produced, which is only present in the reaction path (1). In contrast, reaction (1) produces one molecule of CO per C_2_H_2_ molecule whereas reaction (2) yields twice the amount of CO molecules. Thus, the increase in the amount of CO in the exhaust is a clear indication for the transition from the reaction path (1) to path (2). These results clearly demonstrate that the oxidative dehydrogenation reaction of C_2_H_2_ with CO_2_ proceeds along the reaction (1) below 500 °C, whereas the reaction (2) is kinetically preferrential above 500 °C.

As can be seen in Figure 8, the reaction path also affects the yield of carbon nanotubes produced. At temperatures below 500 °C, the oxidative dehydrogenation reaction follows the reaction (1). When the temperature is increased from 400 to 500 °C, kinetics of the carbon nanotube synthesis thermally enhances, leading to a linear increase of the yield. A maximum is reached at 500 °C where the transition occurs. The slight decrease in the yield above 500 °C can be explained by the fact that twice less carbon atoms are produced along the reaction path (2) despite the thermal enhancement of the reaction kinetics. Consequently, the optimum temperature for the highest yield of carbon nanotubes is at the transition temperature where the reaction path changes from (1) to (2). The same phenomenon has been observed on the different support materials.

It has to be stated that the classical route of thermal decomposition of C_2_H_2_ is limited at low temperatures. Below 600 °C, we obtained only amorphous carbon instead of carbon nanotubes. However, the oxidative dehydrogenation reaction of C_2_H_2_ with CO_2_ results in carbon nanotubes even at 400 °C. Hence, the oxidative dehydrogenation reaction significantly enhances the carbon nanotube growth kinetics and reduces the growth temperature. For different support materials, we observed different optimum temperatures [24]. For carbon fibers, the lowest optimum growth temperature of 400 °C was observed, whereas Al_2_O_3_ and SiC revealed a high optimum temperature of 650 °C. In Table 2 the highest yield growth temperature is listed for 10 different support materials. This variation is assumed to originate from the difference in adsorption strength and configurations of the gas molecules on the surface of the supported catalyst. C_2_H_2_ molecules adsorb most likely on the catalyst surface while CO_2_ preferentially adsorbs on the support and forms a carbonate-like surface adsorbate. The oxidative dehydrogenation reaction could take place at the triple-joint junction, where the interface between the catalyst particle, support and the reaction gases, is located [43].

**Table 2:** The maximum yield temperature obtained for 10 different support materials when the oxidative dehydrogenation reaction is applied.

Support material	Maximum yield growth temperature / °C	Support material	Maximum yield growth temperature / °C
C	400	La_2_O_3_	550
V_2_O_5_	450	Bi_2_O_3_	600
TaB_2_	500	TiO_2_	625
TiN	500	Al_2_O_3_	650
Nb_2_O_5_	500	SiC	650

The oxidative dehydrogenation reaction provides the possibility to grow carbon nanotubes in a broad range of temperatures by selecting the appropriate support material. Moreover, it has to be emphasized that, although the highest yields can be achieved at optimum temperature, the products obtained at lower temperatures also entirely consist of carbon nanotubes with structural characteristics comparable with those produced at the optimum condition. Hence, the oxidative dehydrogenation reaction enables the growth of carbon nanotubes in a broad range of growth temperatures from 300 to 750 °C. Other support materials may exist which have not been tested so far, with an optimum temperature far below the one obtained in this study.

## 3. Experimental Section

### 3.1. Catalyst Preparation

Stoichiometric amount of metal salts (cobalt (II) acetate tetrahydrate and iron (III) nitrate nonahydrate) corresponding to Fe_2_Co composition was dissolved in distilled water. CaCO_3_ particles were subsequently added. The total amount of metal corresponded to about 5wt%, relative to the catalyst support. The precipitation of Fe and Co salts onto the CaCO_3_ particles was subsequently induced by adding a weak base to the solution. Commercially available CaCO_3_ calcite powders were used (Fluka 21060 from Sigma-Aldrich Chemie GmbH and Calofort U from Speciality Minerals). In addition, two drying processes of the catalyst have been employed. At first, the suspension of CaCO_3_ particles covered by Fe, Co salts was dried under vigorous stirring on a hot plate (hereafter called hot drying method). The second process is based on the sublimation of the solvent. The suspension was frozen by dropping into liquid nitrogen. Once collected, it was subsequently placed in a homemade freeze drying chamber. According to the phase diagram of water, sublimation of ice occurred by raising the temperature while the vapor pressure remained below 5 mbar.

### 3.2. Carbon Nanotubes Synthesis and Characterization

The growth of multi-walled carbon nanotubes was performed in our standard fixed bed reactor to accurately measure the acetylene conversion and the production yield. In these conditions, 100 mg of Fe_2_Co supported by CaCO_3_ were introduced in the reactor at 640 °C. Acetylene and argon were fluxed at 1.0 and 45 L/h respectively for 30 minutes. The reactor was subsequently cleaned by fluxing Ar for 10 minutes. It should be noted that the Fe_2_Co catalyst nanoparticles are generated on the CaCO_3_ support by reduction of the Fe and Co salts by the acetylene *in situ*.

For the water assisted growth, carbon nanotube forests were synthesized in a horizontally mounted quartz tube furnace by the water assisted CCVD process. Ethylene (50–400 sccm), as the carbon source, was diluted with argon (100–300 sccm) previously mixed with hydrogen (40–150 sccm). Water was continuously introduced in the reaction chamber by fluxing Ar (5–50 sccm) through a water bubbler. The water content was calibrated by quadrupolar mass spectrometry. Catalyst Fe (1 nm) was deposited on a silicon substrate with 500 nm oxide layer and additional Al_2_O_3_ (10 nm) as top layer. Furnace temperature was raised to 750 °C prior to the growth. One square centimeter pieces of substrate were used for the growth of carbon nanotubes carpets.

For the equimolar C_2_H_2_-CO_2_ reaction, catalyst was prepared by dispersing the support particles in distilled water in which Fe and Co nitrate had previously been dissolved in 2:1 stoichiometry. After precipitation of the Fe_2_Co salt upon drying or by addition of a weak base, the catalyst was collected and dried at 400 °C for 15min to ensure the decomposition of the Fe_2_Co salts. The catalyst was subsequently introduced in the reactor at temperatures varying between 300 °C and 800 °C. An equimolar mixture of C_2_H_2_ and CO_2_ (0.5L/h) was subsequently fluxed with Ar (20L/h) to produce carbon nanotubes.

Samples were characterized by scanning electron microscopy (SEM, Philips XL 30 FEG operated at 30 kV) and transmission electron microscopy (TEM, Philips CM20 and CM200 FEG, both operated at 200 kV).

## 4. Conclusions

Our recent results on the carbon nanotubes grown by CCVD process have been reviewed. We have demonstrated that in the family of Fe_1–x_M_x_ alloys (with M = Co and Ni), the compounds Fe_2_Co and Fe_2_Ni are the most efficient catalysts. Besides the catalyst composition, the catalyst drying process significantly influences the catalyst particle size. The freeze drying process has been identified as favorable in order to avoid agglomeration of catalyst particles during the carbon nanotube synthesis. CaCO_3_ has been found as a support material which actively influences the growth of carbon nanotubes by the CO_2_ produced by its thermal decomposition. The positive effect of CO_2_ has also been demonstrated by adding gaseous CO_2_ into the reactor. The interaction between C_2_H_2_ and CO_2_ significantly promotes carbon nanotube growth and allows the growth of carbon nanotubes on unfavorable substrate materials, as well as at much lower temperatures, rather than via traditional decomposition of C_2_H_2_.

Also the water assisted growth leads to enhanced catalytic efficiency yielding a dense mat of carbon nanotubes. Measurements of bending modulus of individual nanotubes show that the carbon nanotubes originating from the water assisted growth and the equimolar C_2_H_2_-CO_2_ reaction are superior to conventional carbon nanotubes from decomposition of C_2_H_2_. Thus, we can conclude that higher catalytic efficiency results in reduced density of structural density, therefore in enhanced bending modulus. However, the water assisted growth leads to a narrower diameter distribution and consequently to a higher average bending modulus.

The growth of carbon nanotubes still remains complex. However, the recent outstanding results leading to remarkable improvement in the yield of the reaction, as well as in the improved structural and mechanical properties, demonstrate that also novel chemical routes could exist that involve unexplored mechanisms leading to significant improvements of the carbon nanotube growth.
